# Outcome of Clinical Trials with New Extended Half-Life FVIII/IX Concentrates

**DOI:** 10.3390/jcm6040039

**Published:** 2017-03-28

**Authors:** Maria Elisa Mancuso, Elena Santagostino

**Affiliations:** Angelo Bianchi Bonomi Hemophilia and Thrombosis Center, Fondazione IRCCS Ca’ Granda, Ospedale Maggiore Policlinico, Via Pace 9, 20122 Milan, Italy; elena.santagostino@policlinico.mi.it

**Keywords:** hemophilia A, hemophilia B, extended half-life concentrates, long-acting products, pegylation, glycopegylation, Fc fusion, albumin fusion

## Abstract

The development of a new generation of coagulation factors with improved pharmacokinetic profile will change the paradigm of treatment of persons with hemophilia (PWH). The standard treatment in PWH is represented by regular long-term prophylaxis that, given intravenously twice or thrice weekly, is associated with a not-negligible burden on patients’ quality of life. The availability of drugs with improved pharmacokinetic profile may improve prophylaxis feasibility and protection against bleeding episodes. This article summarizes the main results obtained from clinical trials with modified factor VIII (FVIII) and factor IX (FIX) molecules. Published literature on new molecules for replacement treatment in hemophilia A and B was retrieved using PubMed search, and all ongoing clinical trials have been researched via www.clinicaltrials.gov. Such new molecules are usually engineered to have a longer plasma half-life than that which has been obtained by chemical modification (i.e., conjugation with polyethylene glycol, PEG) or by creating recombinant fusion proteins. Results from phase I/III studies in previously treated adults and children are now available for the vast majority of new products, including the results of their use in a surgical setting. On the contrary, trials involving previously untreated patients are still ongoing for all and results not yet available.

## 1. Introduction

In high-income countries, persons with hemophilia (PWH) have an actual life expectancy similar to that of males in the general population [1,2,3,4]. This outstanding result was achieved thanks to the continuous and progressive improvement of replacement therapy with coagulation factor VIII (FVIII) and IX (FIX) first extracted from human plasma and then obtained through recombinant DNA technology. In the frame of this favorable scenario, the optimal form of replacement therapy is long-term regular prophylaxis since childhood, which is able to overthrow bleeding frequency particularly into joints, therefore reducing the development of chronic arthropathy [5,6]. Patients with hemophilia A on prophylaxis require intravenous injections every other day or three times per week, while those with hemophilia B are usually treated twice weekly, owing to the longer half-life of FIX over FVIII (18–20 h instead of 10–12 h) [7,8]. In this light, the development of a new generation of coagulation factors endowed with extended half-life may overall improve the management of PWH and their quality of life by reducing the burden of frequent intravenous injections, the need for central venous lines in children and the loss of adherence to treatment typically seen in adolescents. Moreover, the possibility of maintaining high trough levels allows for uneventful physical activity and permits to effectively cover major surgical procedures with few injections and low factor consumption.

A variety of technologies have been used to obtain the improvement of the pharmacokinetic (PK) profile of recombinant FVIII and FIX including PEGylation, glycoPEGylation, disulfide linkage between light and heavy chain, and fusion with other recombinant proteins as the Fc domain of IgG or albumin [9]. Fc fusion prolongs the half-life by utilizing the neonatal Fc receptor (FcRn), that is constitutively expressed on various cell types and is responsible for endogenous IgG recycling pathway, which protects from lysosomal degradation. Fc-fusion proteins taken up by pino- and/or endocytosis interact with FcRn, which directs the fused coagulation factors to the plasma membrane, recycling them into the circulation [10]. Chemical modification, such as conjugation to hydrophilic polymers as polyethylene glycol (PEG), is able to prolong plasma half-life of coagulation factors thanks to a reduction in the efficiency of the elimination processes (such as renal excretion, receptor mediated uptake and proteolytic degradation), due to steric hindrance by PEG that shrouds the protein. Direct PEGylation can be random or site-specific and may employ PEG moieties of different molecular weight [11]. Other chemical modifications such as the introduction of a disulfide bond between the light and the heavy chains of FVIII allow for a single-chain FVIII molecule with a higher affinity to VWF, thus preventing premature proteolysis and clearance and ultimately extending its half-life to that of VWF that, in animal models, resulted twice that of unmodified rFVIII [12].

This article summarizes the results of the Phase I/III clinical trials conducted with extended half-life FVIII and FIX products in previously treated adult and pediatric patients as well as those of the surgical sub-studies when available. Overall, five new FVIII products and three new FIX products have been developed. Their main characteristics are summarized in Table 1 and Table 2.

Trials involving previously untreated patients (PUPs) are still ongoing for all the new products. Results are not yet available and will not be included in this paper.

Published literature on new molecules for replacement treatment in hemophilia A and B has been retrieved by using PubMed search by using the name of the new molecules (i.e., “Fc fusion FVIII”, “FC fusion FIX”, “BAX855”, “GlycoPEGylated FVIII”, “GlycoPEGylated FIX”, “BAY 94-9027”, “rVIIISingleChain”, and “rIX-FP”). The name of the new molecules were used also to retrieve all ongoing clinical trials whose results are not yet published in full from www.clinicaltrials.gov.

Table 3 summarizes all the study programs, treatment arms of each single study and number of treated patients which will be described in detail throughout the present review.

## 2. Extended Half-Life FVIII Products

### 2.1. Recombinant FVIII Fc (rFVIIIFc) Fusion Protein

rFVIIIFc or efmoroctocog alfa or efraloctocog alfa is a novel engineered recombinant protein that results from the fusion at the genomic level of the FVIII gene with the Fc fragment of IgG1. rFVIIIFc is constituted by a single molecule of B-domain deleted rFVIII covalently linked through its carboxy-terminus to the *N*-terminus of human IgG1 Fc monomer, which forms a disulfide bond with a second Fc monomer during synthesis and secretion from the cells. rFVIIIFc is produced in human embryonic kidney 293 (HEK-293) cells [36].

The phase I/IIa trial (NCT01027377) involved 16 subjects with severe hemophilia A and showed a mean half-life 1.6 times longer compared to that of standard rFVIII, with no difference in the incremental in vivo recovery. rFVIIIFc was well tolerated without serious adverse events related to the drug [13]. None of the subjects developed anti-FVIII inhibitors or anti-rFVIIIFc antibodies [13]. The phase III pivotal trial was an open-label, multicenter, partially randomized study that evaluated the comparative PK of rFVIIIFc and rFVIII and the safety, tolerability, and efficacy of repeated rFVIIIFc dosing for prophylaxis and treatment of acute bleeding in 165 previously treated adults and adolescents (age ≥ 12 years) with severe hemophilia A (NCT01181128; A-LONG) [14]. The study had three treatment arms: arm 1, individualized prophylaxis with 25–65 IU/kg rFVIIIFc every 3–5 days based on PK results to maintain a target trough level between 1 and 3 IU/dL (*n* = 118); arm 2, weekly prophylaxis with a fixed dose of 65 IU/kg rFVIIIFc (*n* = 24); and arm 3, episodic treatment with 10–50 IU/kg rFVIIIFc based on bleeding severity (*n* = 23). All subjects who were already on prophylaxis prior to study entry were enrolled in arm 1, whereas those on episodic treatment had the option to enter arm 1 or to be randomized to enter arm 2 or 3 [14]. Primary study endpoints included annualized bleeding rate (ABR), inhibitor development, and other adverse events. The terminal half-life of rFVIIIFc resulted 19 h, 1.5-fold longer than that of rFVIII. Median ABRs observed in the three treatment arms were 1.6, 3.6, and 33.6, respectively, and in the last three months on study, around 30% of subjects in arm 1 achieved a five-day dosing interval [14]. Across all treatment arms, 87% of bleeding episodes were controlled with a single injection. rFVIIIFc was well tolerated and no subject developed inhibitors [14].

The phase III open-label trial evaluating safety, efficacy and PK of rFVIIIFc in previously treated children aged <12 years with severe hemophilia A (NCT01458106; Kids A-LONG) included 71 subjects, 36 (51%) aged <6 years [15]. The study had a single twice-weekly prophylactic treatment arm with 25 IU/kg and 50 IU/kg rFVIIIFc on Day 1 and 4, respectively. Dose (up to 80 IU/kg) and dosing interval (down to every second day) were adjusted based on PK data and bleeding phenotype [15]. Inhibitor development was the primary endpoint; no subject developed neutralizing antibodies. The mean terminal half-life of rFVIIIFc was 12.7 and 14.9 h in children aged <6 and 6–11 years, respectively with an incremental recovery that was consistent between the two groups and an age-dependent decrease of clearance in older children as already observed with standard rFVIII products [15]. Overall, the median ABR was 1.96 and 0.00 for spontaneous bleeds; 93% of bleeds were controlled with one or two injections, and 46% of children reported no bleeds while on study [15]. At study end 90% of subjects were on a twice-weekly regimen at a median weekly dose of 88 IU/kg that consisted in the reduction of dosing frequency in comparison with pre-study regimen in 74% of children [15].

The long-term safety and efficacy of rFVIIIFc was further evaluated in the frame of an extension trial (NCT01454739; ASPIRE) that included 150 and 61 subjects coming from A-LONG and Kids A-LONG studies, respectively, and who have received more than 100 cumulative exposure days (EDs) to rFVIIIFc [16]. Efficacy and safety results confirmed those obtained in the pivotal trials with a better ABR in patients treated according to an individualized regimen. The vast majority of patients (i.e., 64% of adults and 79% of children) did not change infusion frequency or total weekly prophylactic dose, while 20% and 12% of adults and children reduced their total weekly dose during the extension phase [16].

Both A-LONG, Kids A-LONG, and ASPIRE studies allowed for surgeries and a combined data analysis on those procedures has been recently published [37]. Across all studies, 23 major and 52 minor surgeries were performed in 21 and 41 subjects, respectively. Of the 54 procedures (22 major, 41%) assessed for haemostatic response, all were rated as excellent or good [37]. Surgical procedures were successfully managed by using a median rFVIIIFc dose of 58 IU/kg/infusion with a mean of 1–2 infusions/day.

### 2.2. PEGylated rFVIII Products

BAX 855 or rurioctocog alfa pegol is a randomly PEGylated molecule of full length rFVIII produced in Chinese hamster ovary (CHO) cells. BAX 855 is manufactured by covalently binding a branched 20 kDa PEG moiety to Advate and it is produced through controlled PEGylation in which approximately 60% of the PEG chains are localized to the B-domain [38]. A phase I trial in which safety and PK profile of BAX 855 were assessed in 19 subjects with severe hemophilia A was followed by a pivotal phase II/III trial undertaken in 137 previously treated subjects with severe hemophilia A aged 12–65 years to evaluate efficacy, PK, and safety of BAX 855 administered prophylactically twice-weekly or as episodic treatment (NCT01599819 and NCT01736475; PROLONG-ATE) [17]. The mean half-life of BAX 855 ranged between 14.3 and 16.0 h, resulting 1.4-fold longer than that of Advate. The median ABR on prophylaxis was 1.9 with around 40% bleed-free patients, while 96% of bleeding episodes were successfully treated with 1 or 2 infusions [17]. No inhibitory antibodies were detected. The phase III pediatric trial with BAX 855 (NCT02210091) included 66 previously treated children with severe hemophilia A aged <12 years (32, 48% <6 years) and evaluated immunogenicity, PK profile, efficacy, safety, and quality of life during prophylactic treatment with BAX 855 in this patient population [18]. Children received twice-weekly infusions of 50 ± 10 IU/kg BAX 855 over a six-month period to maintain a trough level above 1% and dose could be escalated up to 80 IU/kg according to predefined criteria [18]. Terminal half-life ranged between 12.7 and 13.9 h confirming the 1.5-fold prolongation as compared with Advate. The total mean ABR was 3.04 with a mean joint ABR of 1.10 and injury-related bleeds were the main contributors to total ABR [18]. Overall, 38% of enrolled subjects had zero bleeds while on study and up to 83% of all bleeds were successfully treated with a single injection. Of the 14 subjects who had target joints at study entry, 10 (71%) had resolution of the target joint. No inhibitors were detected during the study and significant improvement in HRQoL, pain reduction and increased physical activity was also observed [18].

A phase III surgical study with BAX 855 (NCT01913405) initiated in December 2013 is currently ongoing and an interim analysis has been recently published [19]. This first analysis reports on 15 procedures (11 major) in 15 subjects for which overall intra- and peri-operative haemostatic efficacy was rated as “excellent” in all cases and no related adverse events were observed [19]. Surgical procedures were covered on average with 30–35 IU/kg twice daily maintaining a mean trough level well above 50% until post-operative Day 7 [19].

NN7088 or N8-GP or turoctocog alfa pegol is a modified rFVIII molecule obtained by site-directed glycoPEGylation with a 40-kDa PEG moiety attached to a unique O-glycan in the truncated B-domain of turoctocog alfa, a recombinant B-domain truncated rFVIII molecule already employed as replacement therapy in patients with hemophilia A [39]. After thrombin-mediated activation the truncated B-domain (with or without the attached PEG) is cleaved off and generates a rFVIII similar in structure to native activated FVIII. Turoctocog alfa is synthesized in CHO cells and the truncated B-domain consists of a 21-aminoacid linker sequence as previously described [40]. A phase I trial (NCT01205724; Pathfinder™1) was a dose escalation trial including 26 subjects with severe hemophilia A who received one of three dose levels (25, 50 or 75 IU/kg) of N8-GP evaluated by means of pharmacokinetic assessment [20]. N8-GP was well tolerated at either dose and no subject developed inhibitors. The mean terminal half-life was 19 h, representing a 1.6-fold prolongation in comparison with the FVIII products previously used by the patients [20]. The results of a phase III multinational, open-label, non-randomized trial evaluating safety, PK and efficacy of N8-GP used on prophylaxis and for episodic treatment of bleeds in 186 previously treated patients with severe hemophilia A aged ≥12 years have been recently published (NCT01480180; Pathfinder™2) [21]. Of these patients, 175 received regular prophylaxis with 50 IU/kg N8-GP every fourth day with a median ABR of 1.33. Seventy patients (40%) had no bleeds during the trial and overall 84% of bleeding episodes resolved after a single infusion [21]. N8-GP was in general well tolerated. One patient tested positive for a de novo low-titer anti-FVIII inhibitor (1.3 and 1.9 BU/mL on two consecutive samples drawn 14 days apart) after 93 EDs to N8-GP; initially the patient had no signs of clinical impact of the antibody and continued to be treated with N8-GP. However, three months after his rolling over in the extension phase, a high-titer (i.e., 13.5 BU/mL) was detected and the patient was withdrawn from the study [21]. A phase III trial evaluating safety, PK, and efficacy of N8-GP used in prophylaxis in previously treated children with severe hemophilia A aged <12 years is underway, although no longer actively recruiting new subjects ([41], NCT01731600; Pathfinder™5) as well as an actively recruiting surgical study ([41], NCT01489111; Pathfinder™3).

BAY 94-9027 is a site-specific PEGylated B-domain deleted rFVIII molecule with a single dual-branched 60 kDa PEG molecule attached to an engineered cysteine residue [42]. A phase I study (NCT01184820) in 14 subjects with severe hemophilia A demonstrated a terminal half-life of approximately 19 h for BAY 94-9027, 50% longer as compared to sucrose-formulated rFVIII with comparable recovery [22]. Based on PK results, a phase II/III (NCT01580293; PROTECT FVIII), multicenter, open-label, partially randomized study was designed and accomplished to assess the efficacy and safety of BAY 94-9027 in 134 previously treated patients with severe hemophilia A aged 12–65 years [23]. Patients treated on prophylaxis prior to study entry were only eligible for prophylaxis with BAY 94-9027, while those previously treated on demand could choose either to continue episodic treatment or enter the prophylaxis arm. Patients who entered the prophylaxis arm (*n* = 114) received 25 IU/kg BAY 94-9027 twice weekly for a 10-week run-in period. For the following 26 weeks, 86 out of 97 who had ≤1 breakthrough bleed were then randomized 1:1 to receive BAY 94-9027 every 5 (*n* = 43; 45 IU/kg as starting dose) or 7 days (*n* = 43; 60 IU/kg fixed dose), whereas the other 11 plus those 13 who experienced >1 breakthrough bleed continued with BAY 94-9027 30–40 IU/kg twice weekly [23]. Around 25% of patients treated every seven days experienced ≥1 bleed and their dosing frequency was increased to every five days or twice weekly; on the other hand, none of the patients treated every five days increased their dosing frequency [23]. Median ABR on prophylaxis was 4.1 in those treated twice weekly, 1.9 in those treated every five days and 0.96 in those who could maintain a seven-day treatment interval [23]. Up to 91% of bleeding episodes were controlled with one or two infusions. No patient developed anti-FVIII neutralizing antibodies [23]. A phase 3 multicenter, open-label, non-randomized trial is currently underway to investigate efficacy and safety of BAY 94-9027 in previously treated children with severe hemophilia A aged <12 years (NCT01775618; PROTECT VIII Kids) [24]. The study has been recently completed but final results have not been published in full yet. Children started prophylaxis with BAY 94-9027 either 25 IU/kg twice weekly or 45 IU/kg every five days or 60 IU/kg once weekly at investigator’s discretion [24]. As of the latest report, 61 children were treated (32, 52% aged <6 years) and 8/15 (53%) of those who started once weekly needed to increase the dosing frequency to either every five days (*n* = 6) or twice weekly (*n* = 2). The median ABR across all treatment arms was 2.87 for all bleeds, 0 for spontaneous bleeds and 0 for joint bleeds. Almost all bleeds (92%) were successfully treated with 1–2 infusions and 25% of children had no bleeds during the study period [24]. Three children who experienced hypersensitivity symptoms and five who showed loss of efficacy of BAY 94-9026 were withdrawn from the study. No anti-FVIII inhibitors were detected [24].

### 2.3. Single-Chain rFVIII (rVIII-SingleChain)

CSL627 or rVIII-SingleChain is a recombinant single-chain FVIII molecule expressed in CHO cells, designed as a B-domain truncated rFVIII molecule where the light and heavy chains are covalently linked through a disulfide bond, thereby constituting a stable single-chain [43]. Nevertheless, the key thrombin cleavage sites required for its activation are unchanged and the active form structurally comparable with all the other available FVIII products [43]. This novel compound has a higher affinity for VWF as compared with other FVIII molecules, with a binding capacity which is both faster and stronger than that of a full-length unmodified rFVIII [12].

rVIII-SingleChain PK profile, safety and efficacy are under investigation in the frame of a unique clinical trial program designed as a large interconnected series of Phase I/III studies referred to as the AFFINITY clinical trial program. This program includes a first part of studies in adults and adolescents aged 12–65 years with severe hemophilia A (NCT01486927) [25] followed by a Phase III study in pediatric previously treated patients who underwent PK study and received prophylaxis with rVIII-SingleChain ([41], NCT02093897) and an extension study ([41], NCT02172950).

In the Phase I study, 30 adult patients underwent comparative PK study with full-length rFVIII. Patients completing this part entered the Phase II in which they received repeated doses of rVIII-SingleChain on prophylaxis or as episodic treatment, thus allowing to design the treatment dose and schedule for the Phase III study that included overall 173 patients (146 treated on prophylaxis, 54% doses three times per week) and a surgical sub-study in which 16 major surgeries in 13 subjects were evaluated [26]. Preliminary data showed an excellent/good hemostatic efficacy both on prophylaxis and as episodic treatment and a good safety profile of rVIII-SingleChain without inhibitor development [25]. The efficacy of rVIII-SingleChain in the surgical setting was rated as excellent in all cases except one that was rated as good [26].

A first PK analysis has been carried out combining data from treated patients of all ages: these preliminary data show a favorable PK profile for rVIII-SingleChain over full length rFVIII although with a shorter half-life and higher clearance in the pediatric group as expected [44].

## 3. Extended Half-Life FIX Products

### 3.1. Recombinant FIX Fc (rFIXFc) Fusion Protein

rFIXFc or eftrenonacog alfa, is a novel engineered recombinant protein that result from the fusion at genomic level of FIX gene with the Fc fragment of IgG1. rFIXFc is constituted by a single molecule of FIX fused through its carboxy-terminus to the *N*-terminus of a human IgG1 Fc monomer, which forms a disulfide bond with a second Fc monomer during synthesis and secretion from the cells. Activation of FIX is achieved without the need for a cleavable linker between FIX and Fc, at variance with other fusion proteins (see below). rFIXFc is produced in HEK-293 cells [45].

The phase I/IIa trial (NCT00716716) involved 14 subjects with severe hemophilia B and showed a mean half-life 3 times longer than that of unmodified FIX molecule [46]. The incremental in vivo recovery was similar to that of plasma-derived FIX. rFIXFc was well tolerated without serious adverse events. None of the subjects developed anti-FIX inhibitors or anti-rFIXFc antibodies [46]. The phase III pivotal trial was an open-label, multicenter, non-randomized study that evaluated the comparative PK of rFIXFc and rFIX and the safety, tolerability, and efficacy of repeated rFIXFc dosing for prophylaxis and treatment of acute bleeding in 123 previously treated adults and adolescents (age ≥ 12 years) with severe or moderately severe hemophilia B (FIX ≤ 2 IU/dL) (NCT01027364; B-LONG) [27]. Patients enrolled in the study were assigned to one of three treatment groups and 114/123 (93%) were finally included in the efficacy analysis: group 1, weekly prophylaxis with 50 IU/kg rFIXFc as starting dose, eventually adjusted based on PK results to maintain a target trough level between 1 and 3 IU/dL (*n* = 61); group 2, prophylaxis with 100 IU/kg rFIXFc given every 10 days to start and with interval adjusted based on PK results to maintain a target trough level between 1 and 3 IU/dL (*n* = 26); and group 3, episodic treatment with 12–100 IU/kg rFVIIIFc based on bleeding severity (*n* = 27). All subjects who were already on prophylaxis prior to study entry were enrolled in group 1 or 2 at investigator’s discretion whereas those on episodic treatment had the option to enter any of the three treatment groups [27]. A fourth group of treatment pertained those patients candidate to surgery, who entered the surgical sub-study (see below) either directly or from groups 1, 2, or 3 [27]. Primary study endpoints included ABR, inhibitor development and other adverse events. The terminal half-life of rFIXFc resulted 82 h, approximately 4-fold longer than rFIX and the time to reach a trough level of 1 IU/dL was 11.2 days as compared with 5.1 days with rFIX. Median ABRs observed in the 3 treatment arms were 3.1, 2.4, and 18.7, respectively. In group 1 the median dose of weekly prophylaxis was 45 IU/kg and in group 2 the median dosing interval was 12.5 days [27]. Among those subjects in group 2 who were in study for ≥ six months, 54% achieved a dosing interval ≥ 14 days in the last three months on study [27]. Across all treatment arms, 90% of bleeding episodes were controlled with a single injection. rFIXFc was well tolerated and no subject developed anti-FIX neutralizing antibodies [27].

The phase III open-label trial evaluating safety, efficacy and PK of rFIXFc in previously treated children aged <12 years with severe or moderately severe hemophilia B (FIX ≤ 2 IU/dL) (NCT01440946; Kids B-LONG) included 30 subjects [28]. At study entry, all children received prophylaxis at a starting dose of 50-60 IU/kg rFIXFc once weekly with dose and dosing interval adjusted on the basis of PK results and bleeding tendency [28]. The median prophylactic dose was 58.6 IU/kg rFIXFc given once weekly. Overall, median ABR was 2.0 for all bleeds and 0.0 for spontaneous joint bleeds. Ten patients (33%) reported no bleeds during the study [28].

The long-term safety and efficacy of rFIXFc has been further evaluated in the frame of an extension trial (NCT01425723; B-YOND) from which an interim analysis has been recently published [29]. At the time of the interim analysis, the study included 93 and 23 subjects coming from B-LONG and Kids B-LONG studies, respectively and had ≥100 EDs to rFIXFc. Efficacy and safety results confirmed those obtained in the pivotal trials with low ABRs with all prophylaxis regimens and the vast majority of patients maintained the same prophylaxis regimen followed in the pivotal trial [29].

A surgical sub-study was performed in the frame of the B-LONG study as a fourth group of treatment and results were published separately from the pivotal trial [47]. Overall, 14 major procedures were performed in 12 subjects (no minor surgeries were allowed per protocol), being orthopaedic surgery the most common type (*n* = 11/14, 79%). All procedures were rated as excellent but one that was rated as good [47]. The median dose of the pre-operative bolus was 91 IU/kg; most subjects received 2–3 infusions during post-operative Days 1–3 and no subject was dosed every day during the whole peri-operative period (i.e., Days 0–14). The mean nominal dose of rFIXFc administered across all procedures was around 50 IU/kg/day and the median total consumption on Days 1–14 was 432.3 IU/kg (range: 98.6–1084.7) [47].

### 3.2. Recombinant FIX-Albumin Fusion Protein (rIX-FP)

rIX-FP is a recombinant single-chain protein obtained by the fusion of recombinant human FIX with recombinant human albumin connected by a short cleavable linker peptide derived from the endogenous activation peptide of native FIX [48]. The linker is cleaved by the same enzymes that activate wild-type FIX (i.e., FXa or the complex FVIIa/tissue factor) and its cleavage removes the albumin molecule rendering FIX active. rIX-FP is produced in CHO cells and no excipient from animal or human origin is included in the manufacturing process nor in the final formulation. Since albumin is protected from degradation by pH-dependent binding to the neonatal Fc receptor, rIX-FP has an increased circulating half-life as compared with rFIX.

PK, efficacy, and safety of rIX-FP were evaluated in the frame of a large clinical trial program referred to as PROLONG-9FP that included phase I/II/III trials and a surgical study.

Phase I trials (NCT01233440; NCT01361126) included 40 subjects who underwent comparative pharmacokinetics with standard FIX products [49,50]. The mean half-life after a single dose of rIX-FP was 92–95 h and the in vivo incremental recovery was 1.4–1.5, being both parameters greater than those of unmodified FIX products. After a single dose of 25 IU/kg rIX-FP, FIX trough level at seven and 14 post-infusion days were around 5–7 and 2–3 IU/dL, respectively [49,50].

The phase III trial was a multinational, open-label, single-sequence crossover, non-randomized trial in which PK, efficacy and safety of rIX-FP used for treatment and prevention of bleeding episodes was evaluated in 63 previously treated adults and adolescents (aged 12–61 years) with severe or moderately severe hemophilia B (FIX ≤ 2 IU/dL) (NCT0101496274) [30]. In the study the efficacy of seven-day and 14-day prophylaxis regimens was also evaluated. Upon study entry patients were assigned to either prophylaxis (group 1) or on-demand (group 2) treatment based on previous treatment regimen. Group 1 received weekly 35–50 IU/kg rIX-FP for 26 weeks after which it was allowed to switch to 10- or 14-day prophylaxis with 50 or 75 IU/kg rIX-FP, respectively [30]. Group 2 received on-demand treatment during the first 26 weeks followed by 7-day prophylaxis over the following study period. The mean terminal half-life of rIX-FP resulted 102 h, 4.3-fold longer than that of FIX products previously used by patients. With rIX-FP 40 IU/kg weekly and 75 IU/kg every other week, mean troughs levels of 20 and 12 IU/dL were maintained [30]. In subjects who switched from on-demand to prophylaxis regimen a 100% resolution rate of target joints was observed and the median spontaneous ABR was 0 across all prophylactic regimens [30]. Overall, 94% of bleeding episodes were successfully treated with a single injection. No patient developed inhibitors and no safety concerns were observed.

A phase III pediatric trial evaluated PK, efficacy and safety of rIX-FP in 27 previously treated children aged 1–11 years with severe and moderately severe hemophilia B (FIX ≤ 2 IU/dL) (NCT01662531) [31]. All children received prophylaxis with 35–50 IU/kg rIX-FP once weekly. The mean terminal half-life of rIX-FP was 91.4 h, 4.3-fold longer than previous FIX product, confirming results of the pivotal trial. The median spontaneous ABR was 0 with a median prophylactic dose of 46 IU/kg and a median trough level of 13.4 IU/dL. All results were similar in children aged < and ≥6 years [31]. Around 89% of bleeds were successfully managed with one injection. No patient developed anti-FIX inhibitors and no safety issues were observed.

The efficacy and safety of rIX-FP in the surgical setting was assessed in the occasion of 21 procedures performed in 19 patients (including nine major orthopaedic surgeries in eight subjects) in the frame of both the aforementioned phase III trials and published separately [32]. Haemostatic efficacy was rated as excellent or good for all orthopaedic procedures: 8/9 of them were managed with a single pre-operative rIX-FP dose and in 7/9 the first post-operative dose was given more than 24 h after surgery [32]. The median number of infusions administered over the 14-day post-operative period was seven with an overall median concentrate consumption of 375 IU/kg and trough levels persistently well above 50 IU/dL [32].

### 3.3. PEGylated rFIX Products

Nonacog beta pegol or N9-GP is a recombinant FIX molecule with a prolonged half-life obtained by site-directed glycoPEGylation so that a branched 40 kDa PEG moiety is attached to the activation peptide of FIX at two possible sites (Asn157 or Asn167). However, N9-GP is mainly mono-PEGylated with an equal distribution of the two possible PEGylation sites. GlycoPEGylation is obtained enzymatically and terminal sialic acids on the N-glycan structures of rFIX are replaced with sialic acid conjugated to PEG. Upon physiological activation, the activation peptide (with the attached PEG) is cleaved off yielding the wild-type activated FIX. The rFIX part of N9-GP is synthesized in CHO cells and the amino acid sequence is identical to that of the unmodified rFIX products [51].

Data from the phase I trial (NCT00956345; Paradigm™1) obtained from 16 previously treated subjects who received one dose of their usual FIX product followed by the same single dose of N9-GP showed a five-fold prolongation of the half-life with the latter allowing for once-weekly prophylaxis [52]. Additionally, the incremental in vivo recovery for N9-GP was 94% and 20% greater compared to both recombinant and plasma-derived FIX products, respectively [52]. N9-GP was well tolerated by all subjects but one, who showed a hypersensitivity reaction soon after the injection of N9-GP that caused his withdrawal from the trial. None of the subjects (including the latter) developed inhibitors. The phase III trial conducted in adult and adolescent patients was a multinational, randomized, single-blind study aimed at investigating PK, safety, and efficacy of N9-GP in 74 previously treated patients with hemophilia B (FIX ≤ 2 IU/dL) (NCT01333111; Paradigm™2) [33]. Patients received either episodic or prophylactic treatment based on previous regimen and preference; those who were treated on prophylaxis were randomly assigned 1:1 in a blinded fashion to receive either 10 IU/kg or 40 IU/kg N9-GP once weekly for 52 weeks [33]. The median ABRs were 2.9, 1.0 and 15.6 in the 10 IU/kg, 40 IU/kg and on-demand treatment arm, respectively [33]. In the 40 IU/kg prophylaxis arm 67% of patients had resolution of target joints at variance with only 7% in the 10 IU/kg arm. No patients developed inhibitors and no safety concerns were identified. Another phase III trial investigated safety, efficacy and PK of N9-GP in 25 previously treated boys with hemophilia B (FIX ≤ 2 IU/dL) aged ≤12 years (NCT01467427; Paradigm™5) [34]. All children received prophylaxis with 40 IU/kg N9-GP once weekly for 50 EDs. The median ABRs were 1.0 in the total population, 0.0 in the 0–6 years group and 2.0 in the 7–12 years group with an estimated mean steady-state FIX trough levels of 15.3 and 19.0 IU/dL in the 2 age groups, respectively [34]. For those children who were already in prophylaxis prior to study entry the ABR on study was reduced as compared with the historical one (1.38 vs. 2.51, respectively). Overall, 86% of bleeds were solved after one single injection. No patient developed inhibitors and no safety concerns were identified [34].

The efficacy and safety of N9-GP in the peri- and post-operative period was evaluated in the frame of a multicentre, open-label trial that included 13 major procedures in 13 patients (NCT01386528; Paradigm™3) [35]. All patients received a pre-operative bolus of 80 IU/kg N9-GP followed by fixed doses of 40 IU/kg repeated at investigator’s discretion. Intra-operative efficacy was rated excellent (10/13, 77%) or good; none of the patient received a second N9-GP injection on day of surgery and the median number of post-operative doses was 2.0 on Days 1–6 and 1.5 on Days 7–13 with a total median consumption of 126.1 IU/kg over two weeks. No severe adverse events, including inhibitor development, were observed [35]. Two patients developed wound haematomas that did not require surgical evacuation and for which only one patient received an additional N9-GP dose.

## 4. Conclusions

As shown by phase III clinical trials, these improved products should be able to more widely space the doses of infused factors in the frame of regular prophylaxis. This goal is widely achieved with FIX products because the degree of prolongation of FIX half-life was as great as 4–5 times. Long-acting products permit to maintain plasma levels of FIX well above 10 IU/dL with once weekly injections or injections every two weeks. This would be a significant step forward in terms of sparing venous access and better protect patients from breakthrough bleeds, also allowing for a more active lifestyle. Also the rate of control of bleeding episodes resulted high with a success rate of 80%–90% with a single infusion.

On the other hand, the current status of new FVIII products is at the moment less exciting than that of FIX, however still interesting in some groups of patients. The available data indicate that the prolongation of the half-life in comparison to that of wild-type factor is smaller, no more than 1.5–1.8 times. Perhaps the modest prolongation of the FVIII half-life would not ameliorate significantly the burden of infusions in patients already treated successfully 2–3 times weekly. However, switching from an every other day to an every fourth day regimen would indeed facilitate prophylaxis in patients with poor venous access and perhaps increase patient compliance.

Overall, for both new FIX and FVIII products preliminary data from phase III trials suggest that treatment tailored according to pharmacokinetic data provide better outcomes as compared to fixed therapy regimens in terms of reduction of bleeding frequency and, maybe, factor consumption. In fact, in all trials, rather high doses were used in the fixed regimens to ensure higher trough levels as compared with the individualized regimens.

With respect to the safety profile, no major issues have been raised until now, but long-term post-licensing surveillance studies are warranted to keep track of any potential side-effect on a larger scale. Whether or not the different mechanisms of half-life prolongation imply issues of long-term safety (i.e., fusion technology versus PEGylation) is not clear yet. Some concerns have been raised on the possible accumulation and consequent side effects of long-term exposures to PEG, since previous uneventful experiences with other PEGylated drugs refer to short-term exposures. Crucial issues are immunogenicity in PUPs and costs; the first will be clarified by the results of “ad hoc” designed clinical trials currently ongoing.

Pertaining to costs at the moment, not all products are available on the market and, ideally, the pricing of these new products should be based on the simple equation of multiplying that of the currently available products by the doses of the latter that are spared owing to the longer–half life. It is doubtful that manufacturers will accept this, in consideration of the costs that they had to meet for high quality research and development. However, the current global economy crisis demands that the cost of treatment of PWH does not increase beyond the already high threshold, and indeed, in some European countries, some of these new products have entered the market with a price/Unit that almost equals that of standard products in terms of cost of annual course of therapy.

## Figures and Tables

**Table 1:** New extended FVIII products.

Product/Company	Reference	Technology	Terminal Half-Life	Current Status
Elocta^®^/Eloctate^®^, Biogen Idec/Sobi	[13,14,15,16]	Fusion protein with the Fc fragment of IgG1 (rFVIIIFc)	19 h	Marketed in USA, Canada, Europe
Adynovate^®^, Baxalta/Shire	[17,18,19]	Random PEGylation (BAX 855; 20 kDa)	14–16 h	Marketed in USA, Japan and Switzerland
NN7088, Novo Nordisk	[20,21]	Site-specific glycoPEGylation (N8-GP; 40 kDa)	19 h	Extension phase clinical trial
BAY 94-9027, Bayer	[22,23,24]	Site-specific PEGylation (K1804C PEGylation; 60 kDa)	19 h	Extension phase clinical trial
Afstyla^®^, CSL Behring	[25,26]	Single chain rFVIII (CSL627)	Not available	Marketed in USA

**Table 2:** New extended half-life FIX products.

Product/Company	Reference	Technology	Terminal Half-Life	Current Stage of Clinical Research
Alprolix^®^, Biogen Idec/Sobi	[27,28,29]	Fusion protein with the Fc fragment of IgG1	82 h	Marketed in USA, Canada
Idelvion^®^, CSL-Behring	[30,31,32]	Fusion protein with albumin	102 h	Marketed in USA, Japan, Europe
N9-GP, Novo Nordisk	[33,34,35]	Site-specific glycoPEGylation	93 h	Extension phase clinical trial

**Table 3:** Study programs with new FVIII and FIX molecules.

Study Program	Molecule	Treatment Arms	*N* of Patients	ABR
*A-LONG*	rFVIIIFc		118	1.6
12–65 years		***1***. 25–65 IU/kg every 3–5 days to maintain FVIII trough 1–3 IU/dL (tailored prophylaxis)	24	3.6
		***2***. 65 IU/kg once weekly (fixed regimen)	23	33.6
		***3***. 10–50 IU/kg on demand		
<12 years		25 IU/kg on Day 1 + 50 IU/kg on Day 4	35 (6–11 years)	0.0
		(dose and dosing interval were adjusted based on PK and bleeding tendency)	36 (<6 years)	1.96
*PROLONG-ATE*	BAX 855			
12–65 years		45 ± 5 IU/kg twice weekly	137	1.9
<12 years		50 ± 10 IU/kg twice weekly	66	3.04
*Pathfinder™*	N8-GP			
12–65 years (Pathfinder™2)		50 IU/kg every fourth day	175	1.33
<12 years (Pathfinder™5)		ongoing	ongoing	ongoing
*PROTECT FVIII*	BAY 94–9027			
12–65 years		25 IU/kg twice weekly for 10 weeks (run-in period) than:		
		***1***. 30–40 IU/kg twice weekly (≤ 1 bleed in the run-in period)	11	1.9
		***2a***. 45 IU/kg every 5 days * (≤ 1 bleed in the run-in period)	43	1.9
		***2b***. 60 IU/kg once weekly * (≤ 1 bleed in the run-in period)	43	3.9
		***3***. 30–40 IU/kg twice weekly (> 1 bleed during the run-in period)	13	4.1
		* upon randomization		
		On demand	20	23.4
<12 years		***1***. 25 IU/kg twice weekly °	ongoing	ongoing
		***2***. 45 IU/kg every 5 days °	ongoing	ongoing
		***3***. 60 IU/kg once weekly °	ongoing	ongoing
		° at investigator’s discretion		
*AFFINITY*	rVIII-SingleChain			
12–65 years		ongoing	173	ongoing
<12 years		ongoing	ongoing	ongoing
*B-LONG*	rFIXFc			
12–65 years		***1***. 50 IU/kg once weekly; dose adjusted on PK to maintain FIX 1–3 IU/dL	61	3.1
		***2***. 100 IU/kg every 10 days; interval adjusted on PK to maintain FIX 1–3 IU/dL	26	2.4
		***3***. 12–100 IU/kg on demand	27	18.7
<12 years		50–60 IU/kg once weekly	30	2.0
*PROLONG-9FP*	rIX-FP			
12–65 years		***1***. 35–50 IU/kg once weekly for 26 weeks; then:	40	0.0
		50 IU/kg every 10 days or 75 IU/kg every 14 days		
		***2***. on demand for 26 weeks followed by 35–50 IU/kg once weekly	23	19.2
<12 years		35–50 IU/kg once weekly	27	0.0
*Paradigm™*	N9-GP			
12–65 years (Paradigm™2)		***1***. 10 IU/kg once weekly	30	2.9
		***2***. 40 IU/kg once weekly	29	1.0
		***3***. on demand	15	15.6
<12 years (Paradigm™5)		40 IU/kg once weekly	25	1.0

* Upon randomization; ° at investigator’s discretion.
